# Sternal-Wound Infections following Coronary Artery Bypass Graft: Could Implementing Value-Based Purchasing be Beneficial?

**DOI:** 10.36469/jheor.2020.13687

**Published:** 2020-08-18

**Authors:** Dominique Brandt, Maximilian Blüher, Julie Lankiewicz, Peter J. Mallow, Rhodri Saunders

**Affiliations:** 1 Health Economics & Clinical Outcomes Research, Xavier University, Cincinnati, OH, USA; 2 Coreva Scientific, Königswinter, Germany; 3 Cardinal Health, Dublin, OH, USA

**Keywords:** SWI, health care costs, hospital costs, LOS, coronary artery bypass graft

## Abstract

**Background/Objectives:**

Sternal-wound infections (SWIs) are rare but consequential healthcare-healthcare-associated infections following coronary artery bypass graft surgery (CABG). The impact of SWIs associated on the cost of health care provision is unknown. The aim of this study was to quantify the burden of CABG-related SWIs across countries with mature health care systems and estimate value-based purchasing (VBP) levels based on the local burden.

**Methods:**

A structured literature review identified relevant data for 14 countries (the Netherlands, France, Germany, Austria, the United Kingdom, Canada, Italy, Japan, Spain, the United States, Brazil, Israel, Taiwan, and Thailand). Data, including SWI rates, CABG volume, and length of stay, were used to populate a previously published Markov model that simulates the patient’s CABG-care pathway and estimates the economic (US$) and care burden of SWIs for each country. Based on this burden, scenarios for VBP were explored for each country. A feasible cost of intervention per patient for an intervention providing a 20% reduction in the SWI rate was calculated.

**Results:**

The SWI burden varied considerably between settings, with SWIs occurring in 2.8% (the United Kingdom) to 10.4% (the Netherlands) of CABG procedures, while the costs per SWI varied between US$8172 (Brazil) to US$54 180 (Japan). Additional length of stay after SWI was the largest cost driver. The overall highest annual burden was identified in the United States (US$336 million) at a mean cost of US$36 769 per SWI. Given the SWI burden, the median cost of intervention per patient that a hospital could afford ranged from US$20 (US$13 to US$42) in France to US$111 (US$65 to US$183) in Japan.

**Conclusions:**

SWIs represent a large burden with a median cost of US$13 995 per case and US$900 per CABG procedure. By tackling SWIs, there is potential to simultaneously reduce the burden on health care systems and improve outcomes for patients. Mutually beneficial VBP agreements might be one method to promote uptake of novel methods of SWI prevention.

## BACKGROUND

Healthcare-associated infections (HAIs), which are infections contracted in hospital while in care for another condition, represent a significant clinical and economic burden to hospitals and their patients.^1^ Collecting data on HAIs is complex, and currently there are no established standards for systematically reporting HAIs, making it difficult to estimate the global burden of HAIs.^1^,^2^ According to the WHO, HAIs affect 5.1% to 19% of hospitalized patients worldwide, and the prevalence and nature of HAIs are closely linked to economic development and quality of care. In the United States, the annual economic burden of HAIs has been estimated to range between US$28 billion and US$45 billion, with HAIs affecting 2 million patients and causing 90 000 deaths yearly.^3^ Costs vary from prolonged impatient stay, long-term disability, lost productivity, and death.^1^,^4^ Becoming more pertinent are the additional use of antibiotics and development of antimicrobial resistance despite increased emphasis on handwashing, sterilization, and terminal cleaning.^1^,^4^

Many developed health care systems have started to move away from fee-for-service payment models toward payment-for-performance, in hope of improving patient care and curbing costs.^5^–^7^ In the United States, the Centers for Medicare & Medicaid (CMS), which oversees United States federal health care programs, is now denying payment for treatment of certain HAIs.^8^ Since 2012, CMS has been applying a hospital value-based purchasing (VBP) model, where acute-care hospitals are paid according to their performance.^9^ Hospitals are compared to benchmarks defined for four domains: clinical care; person and community engagement; safety; and efficiency and cost reduction. Hospitals ranking in the worst performing quartile are sanctioned through a 1% reduction in payment.^10^ Furthermore, payments are denied for readmission following certain procedures, such coronary artery bypass graft surgery (CABG).^9^,^11^ Similar measures have also been undertaken by private payers in Australia, where Medibank introduced quality of service requirements in 2015 when payments for additional costs due to HAIs are denied.^12^ In the United Kingdom, readmissions within 30 days due to a surgical-site infection are not reimbursed.^13^

Shifting (at least partially) the cost impact from payers to providers may reduce national spending on health care, but it puts additional strain on hospitals and their budgets. Such performance penalties are complex to forecast and challenging to take into account in the budget. With a poor performance rating also impacting patient confidence, hospitals are searching for affordable ways to reduce HAIs.^14^–^17^

Sternal-wound infections (SWIs), which can occur after cardiac surgery, are a major and well-defined contributor to the burden of HAIs.^18^ Unlike most HAIs and surgical-site infections, the definition of SWIs is generally more consistent across health care systems. About 0.5% to 8% of patients are at risk of developing a superficial SWI in the pectoralis fascia, the subcutaneous tissue, and the skin.^19^–^21^ Superficial SWIs are often easily treatable with topical wound care and antibiotics.^22^ However, more severe infections, such as mediastinitis or deep SWIs (DSWIs), are associated with high morbidity and mortality.^23^ According to a 2015 review by Cotogni et al, 0.5% to 6.8% of cardiac surgery patients develop DSWIs, with in-hospital mortality rates ranging from 7% to 35%.^20^

SWIs come at a great cost with increased length of stay (LOS), high readmission rates, and reduced patient quality of life, which can fall below presurgical levels.^24^ CABG is one of the most commonly performed cardiac surgeries. It is a universally accepted, highly reported, and complex surgical procedure. Thus, it is well suited for comparisons of global SWI rates.

CABG is a suitable setting for exploring the introduction of new interventions that could reduce the burden of SWIs. The introduction of beneficial interventions can, however, be impaired due to cost concerns. VBP involves a (generally low) base purchase price and a final price that is calculated from the value it provides to the purchaser—in this case hospitals—after extended use. VBP helps to ensure low financial risk to hospitals. Any additional savings derived from the new intervention could be shared between the purchaser and seller. If no benefit is derived, then the seller receives no additional payment beyond the base purchase price.

The focus of this study was to evaluate the global burden of SWIs after CABG using comparable mature health care systems. The premise was to provide hospitals with a foundation when considering strategies to reduce their incidence of SWIs and corresponding costs in a VBP model.

## METHODS

### Setting

This study assessed the burden of SWIs after CABG in countries with mature health care systems, as defined by the 2017 Global Access to Healthcare report of The Economist Intelligence Unit.^25^ The report identified 15 countries with mature health care systems according to their Global Access to Healthcare Index: the Netherlands, France, Germany, Austria, the United Kingdom, Canada, Cuba, Italy, Japan, Spain, the United States, Brazil, Israel, Taiwan, and Thailand.

### Model Structure

We defined the burden of SWIs in three ways: (1) additional length of hospital stay, in the intensive care unit (ICU) or general ward (GW); (2) readmissions; and (3) additional cost of hospital care. To estimate the burden in each country, a Markov model with states representing the CABG-care pathway was used. This Markov model was adapted from a US-specific model published by Saunders and Lankiewicz.^26^ Here the model was generalized for countries with similarly mature health care systems.

The analysis was completed as follows: A patient entered the model at surgery and was then taken to the ICU, where they received mechanical ventilation. During the time in ICU, the patient recovered sufficiently to be taken off the ventilator, discharged to the GW, and then discharged home. At all times, the patient was at risk of developing either a superficial SWI or a DSWI and dying. The cumulative incidence of SWI over time postsurgery was taken from Lankiewicz et al.^27^ The cumulative incidence curve was modeled using a dose–response Hill curve (Cumulative SWI = α + (θ*x*^η^)/(K^η^ + *x*^η^)), found to be most representative of the input data using CurveExpert Pro (Hyams Development, Chattanooga, TN), with alpha 2.96_x10_^−30^, theta 6.93, eta 1.08, and kappa 23.73. The curve was assumed to be consistent across countries, but it was adjusted up or down by a percentage factor for each country so that it provided the correct incidence of SWIs. Specifically, if country-specific data had an SWI incidence of 2.4% at 30 days, and the cumulative incidence curve gave an incidence of 4.8% at 30 days, then each value derived from the cumulative incidence curve would be multiplied by 0.5 (2.4/4.8). Probabilities of progression through the different stages of the modeled pathway were country specific, as were the additional LOS associated with SWIs, the cost of ICU and GW days, and the number of CABG procedures.^27^ All costs were converted to 2017 US$ using market exchange rate values at mid-range for the year.

### Model Inputs

To identify country- and hospital-level data to populate the model, a review of the literature was performed for CABG surgeries, SWIs, DSWIs, SWI follow-up, LOS, and daily costs of ICU and GW. The literature review was performed in PubMed and Google Scholar by DB, MB, and PJM, with extracted country data checked for accuracy by a second author: either RS, MB, DB, or PJM. Countries in which at least 5 (of the 10 country-specific) parameters were identified were included in the model. Missing data for the retained countries were calculated using the median and interquartile ranges (IQRs) of values from countries for which data were available. In values for which more than one study was found, the midpoint was used. The robustness of the model was assessed using probabilistic sensitivity analysis with 52 iterations per country.

### VBP

VBP levels were estimated using three assumptions: (1) no purchasing hospital would commit all the estimated cost burden to preventative measures; (2) available funds would be split over multiple interventions; and (3) any purchase agreement would include a cost of intervention per patient (CIPP) and a share in savings generated from reduced SWIs. The last item is the VBP, which is paid out only if the intervention meets its stated target. The potential purchase CIPP and VBP were calculated using the following formulas:

CIPP=(1-RRSWIINT)·CSWI·rSWI·SVBPPUR100NVBPINTVBP=(rSWI·NPT·CSWI·(1-RRSWIINT))-(CIPP·NVBPINT·NPT)(100·NVBPINT)SVBPSAV

where for the CIPP formula, ${\text{RR}}_{\text{SWI}}^{\text{INT}}$ is the relative risk of SWI events when using the intervention; C^SWI^ is the cost per SWI; ${\text{S}}_{\text{VBP}}^{\text{PUR}}$ is the share of the SWI cost allocated to purchase of interventions under the VBP scheme; and ${\text{N}}_{\text{VBP}}^{\text{INT}}$ is the number of interventions being considered. When calculating the VBP, r^SWI^ is the SWI rate at the hospital (inpatient events plus readmissions); N^PT^ is the number of patients in the target population; and ${\text{S}}_{\text{VBP}}^{\text{SAV}}$ is the share of the cost savings being committed if an intervention meets its target under the VBP scheme.

### Scenario Analysis

The following scenarios were considered: (1) 50% of the cost burden is made available for VBP of two interventions with a 15% savings share on success, and (2) 30% of the cost burden is made available for VBP of two interventions with a 25% savings share on success. All CIPP and VBP calculations assumed a hospital performing 1000 CABG procedures per year, were performed on the results of the probabilistic sensitivity analysis, and are presented as the median and range.

## RESULTS

**Figure 1:**
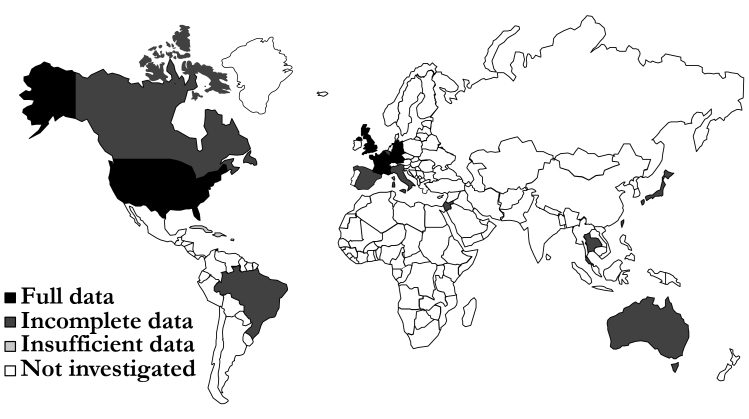
Data Availability for the Target Countries Country color indicates the level of data availability, with countries highlighted in black having country-specific data for all 10 parameters. Those in dark gray were missing one, two, or three parameters; Cuba (light gray) had no data identified. Countries in white were not investigated in our analysis.

**Figure 2:**
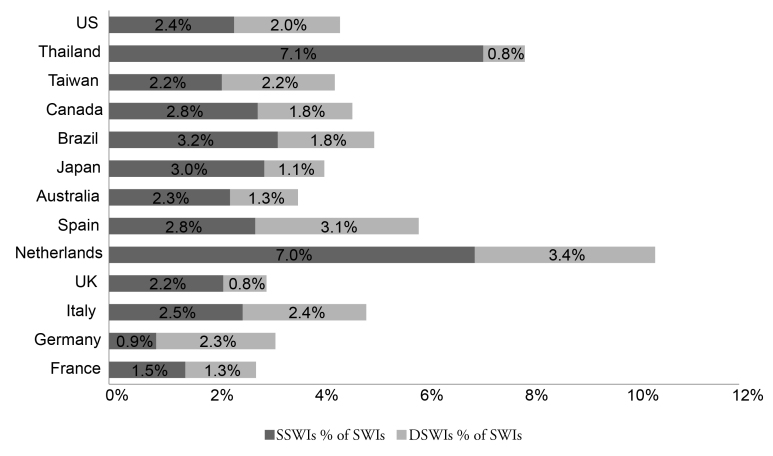
Prevalence of Superficial and DSWIs in Analyzed Countries Abbreviations: CABG, coronary artery bypass graft surgery; DSWI, deep SWI; SWI, sternal-wound infection; SSWI, superficial SWI. Results for Israel are not shown in the graph as information on DSWIs was not available. Israel had a total of 3.6% of SWIs per CABG procedure.

Required data were identified for France, Germany, the Netherlands, the United Kingdom and the United States (Figure 1). One parameter was missing for Australia, Canada, Italy, Japan, Spain, and Taiwan**;** two are missing for Israel and Brazil**;** and three are missing for Thailand. No data were identified for Cuba. Extracted data showed that there was a high variability in prevalence of CABG procedures between countries. Germany had the highest rate with 61.4 CABG procedures per a population of 100 000, whereas Taiwan had the lowest with 6.4 per 100 000.^28^,^29^ Similarly, high variability in relative additional LOS was associated with superficial SWIs, ranging from 2 days in Spain to 49 days in Japan.^28^,^30^ The prevalence of deep sternal wounds, which impacted LOS and readmission rates, ranged between 3.4% (the Netherlands) and 0.8% (the United Kingdom and Thailand, Figure 2). The data used in the analysis can be found in the Supplementary Material, Table S1.

The model estimated the total burden of CABG-related SWIs in target countries to be US$557.7 million, with 60% of the burden (US$336.0 million, Table 1) located in the United States. Taiwan had the lowest burden, estimated at US$1.5 million. The cost per SWI was highest in Japan (US$54 180) and lowest in Brazil (US$8172, Table 1). The median cost per SWI across the analyzed countries was US$13 995 (IQR US$8172; US$23 590). When the total SWI burden was normalized by the procedure volume, the burden was the highest in Japan (US$2795 per procedure; Table 1) and in the United States (US$2113) and the lowest in the United Kingdom (US$436) and France (US$440). The model showed when SWI costs were estimated per CABG procedure, France had the lowest SWI cost per procedure and Japan had the highest (Table 1). In Japan and the United States, costs per day for ICU and GW care were much higher than in the other countries in the model.

**Figure 3:**
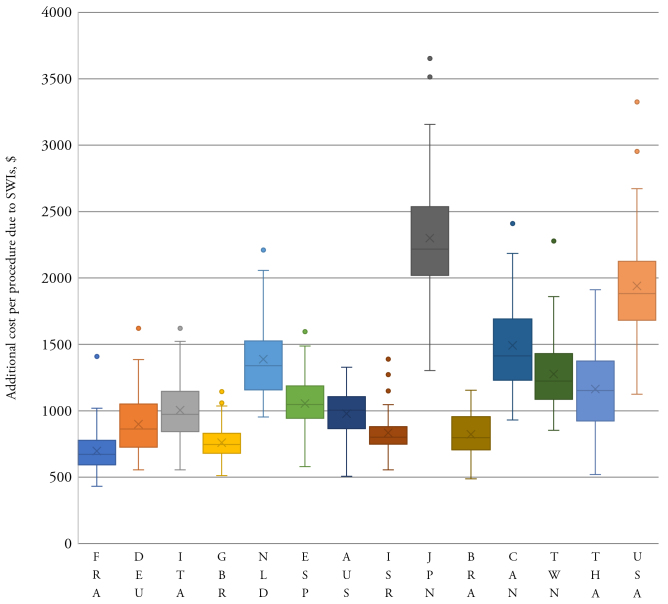
Estimated Cost of SWIs Per CABG in Evaluated Countries Abbreviations: AUS, Australia; BRA, Brazil; CABG, coronary artery bypass graft surgery; CAN, Canada; DEU, Germany; ESP, Spain; FRA, France; GBR, Great Britain; ISR, Israel; ITA, Italy; JPN, Japan; NLD, the Netherlands; SWI, sternal-wound infection; THA, Thailand; TWN, Taiwan; USA: the United States. The cost (in 2017 US$) that SWIs add to each CABG procedure is depicted as a box plot for each country. The shaded box indicates the interquartile range, with the whiskers being the standard deviation. Additional points plotted singularly are considered outliers. Within the shaded box, the line represents the median value, and the cross represents the mean. The box plot is informed by 52 simulations.

The results were generally robust to changes in model parameters during sensitivity analysis. The estimated cost of a SWI per CABG was consistently higher in Japan and the United States compared to other countries analyzed (Figure 3). The lower bound of the IQR for these two countries was more than the upper bound for all other countries. Even at the lowest estimate, the overall cost and resource use burden of SWIs following CABG in the United States was far greater than in any other country (Table 2). The total median burden over all analyzed countries was: US$529 million, 57 994 ICU days; 321 973 GW days; and 9418 readmissions.

The potential for VBP to help combat postsurgical infections was assessed based on the burden of SWIs following CABG. We provide a worked example for Spain, where the cost of an SWI was estimated at US$12 008 (C^SWI^, Table 1). A Spanish hospital with an inpatient SWI rate of 4% and with 1.6% readmissions, total 5.6% (r^SWI^ = 0.056)—that was looking to invest 30% of potential savings (${\text{S}}_{\text{VBP}}^{\text{PUR}}=30$) in three interventions (${\text{N}}_{\text{VBP}}^{\text{INT}}=3$), targeting a 25% reduction in $\text{SWIs}\;({\text{RR}}_{\text{SWI}}^{\text{INT}}=0.75)$—would have: CIPP = ((1 – 0.75) · 12 008 · 0.056 · 30/100)/3 = US$16.81. Given the fact that the hospital performs 1200 CABG procedures per year (N^PT^ = 1200) and is offering a further 30% of any savings as a $\text{VBP}\;({\text{S}}_{\text{VBP}}^{\text{SAV}}=30)$, the potential per intervention VBP=(0.056·1200·12008·(1-0.75))-(16.81·3·1200)(100·3)30=US$14121.84. If successful, the hospital would realize savings of US$87 036 per year.

Under scenario 1, the median (range) CIPP for an intervention reducing the SWI rate by 20% was from US$34 (US$22–US$70) in France to US$111 (US$65–US$183) in Japan (Table 3). If the intervention succeeded in reducing the SWI rate by 20%, a median VBP of between US$5044 (France) and US$16 629 (Japan) would be received; the median hospital saving would be between US$57 165 (France, US$57 per patient) and US$188 460 (Japan, US$188 per patient).

Under purchasing scenario 2, the median CIPP was lower than in scenario 1. The CIPP ranged from US$20 (US$13–US$42) in France to US$67 (US$39–US$110) in Japan (Table 3). The VBP was generally higher, as was the hospital saving, if interventions met their target under scenario 2. With a 20% reduction in the SWI rate, the median hospital savings ranged from US$70 616 (US$71 per patient) in France to US$232 804 (US$232 per patient) in Japan (Table 3).

## DISCUSSION

SWIs after CABG pose a heavy clinical and economic burden on hospitals. Our study found that countries with mature health care systems incurred a median cost of US$13 995 (IQR US$8172; US$23 590) per SWI. Costs varied according to the individual country care pathways; in Japan and Australia, costs incurred were largely due to the extended LOS to treat DSWIs (averages of 66 and 53.2 days respectively).^31^,^32^ In the United States, in addition to the high cost of care, readmission costs were 10 times higher than in France and Canada and three times higher than in Germany.

The cost per SWI was the highest in the United States at US$36 768 per case, similar to hospital-acquired *Clostridium difficile* infection, which has been reported to be US$34 157 (90% CI: US$33 134, US$35 180).^33^ As CMS moves forward with the Hospital-Acquired Condition Reduction Program, thereby reducing payments to the worse-performing quartile of hospitals with regard to their hospital-acquired conditions score, hospitals may choose to focus on infection reduction measures. Similar systems are in place around the globe, and there will be debate within hospitals as to whether reduction should focus on specific areas with achievable goals or on hospital-wide systems.

There has been published success in reducing severe post-CABG infections, with studies demonstrating substantial reduction in DSWIs. Implementing a quality improvement process, a regional US medical center managed to achieve close to zero DSWIs.^34^ The authors used a bundled approach that included a multidisciplinary collaboration and a change in care pathways. The interventions included standardization of processes, new suture technique with braided triclosan-coated suture, silver-coated midsternal dressing, disposable electrocardiogram leads and wires, an insulin infusion protocol, chlorhexidine mouthwash, preoperative vancomycin, preoperative bath, and patient education.^34^ Similar achievements were made in Israel with an implemented wound-care protocol, the use of chlorhexidine–alcohol, and the exclusion of obese and diabetic women from bilateral internal thoracic artery graft.^35^ In both studies, it was a combination of changes that lead to success.

Such extensive updates of the care pathway may not be feasible in all institutions. Any intervention to reduce the burden of SWIs would, however, be of benefit if it were priced appropriately. Studies have shown that a single intervention can be effective at reducing infection rates, such as introducing 24-hour IV antibiotic prophylaxis,^36^ local gentamicin sponges,^37^,^38^ interlocking figure-eight and nitinol flexigrip closure,^36^,^39^,^40^ and single-use ECG cables and leads.^26^,^34^ Using equations provided in the methods section and our estimates of the SWI burden, providers can calculate how much they may pay on a per patient basis for implementing one or more of these interventions. Given the high cost burden of SWIs, assigning 30% of the estimated savings toward purchasing two new interventions resulted in a viable cost of between US$11 (lowest estimate, France) and US$110 (highest estimate, Japan) per patient. The lowest of these costs per patient likely already covered the standard purchase cost for a number of available options.

With VBP, however, the simple purchase price is not the end of the story. Providers need to monitor and track their progress on infection rates, so that the benefits of value-based and risk-sharing contracts can be leveraged. Sellers also need to remain engaged, promoting continuing education and correct use of the product to see any additional value returned.

## LIMITATIONS

Our results were drawn from a simulation model and do not capture all aspects and subtleties of real-life care. The probabilities of moving from one health state to another were taken from published peer-reviewed studies, and for the feasibility of the model it was assumed that average patient characteristics and risk factors for developing an SWI were the same between countries. Risk factors modeled included morbid obesity (>35 kg/m^2^) and presence of diabetes; less prevalent comorbidities—such as chronic obstructive pulmonary disease, kidney disease, or peripheral vascular disease—were not included.^20^ There was uncertainty about some model parameters, with limited data available for numbers of days to treat SWIs (3 out of 14 countries had missing values) and DSWI (5 out of 14 countries), as well as for cost per day for care on the GW (3 out of 14 countries had missing values). The latter may be due to analyzed countries having their own hospital data collection systems only intended for policy implementation purposes, and our search was limited to the English language.^41^,^42^ Finally, our model assumed an equivalent care pathway in all settings, but care delivery likely varies (if only minorly) among health care systems, hospitals, and care units.^6^,^43^–^45^

## CONCLUSIONS

SWI and DSWI have a high cost, with a median of US$13 995 per case and US$900 per CABG procedure. The overall cost was largely due to increased cost of care and LOS. The cost of readmissions was also a considerable concern. As hospitals are becoming more and more accountable for their outcomes, they may need to rethink care delivery pathways and invest in new procedures and equipment. Reduction of DSWIs is possible but requires investment in both process and infection prevention products.

## IMPLICATIONS

SWI is an area of care where VBP could be implemented, making care improvement possible with limited financial risk to hospitals.

## Supplementary Information



## Figures and Tables

**Table 1:** Burden of CABG-Related SWIs by Country with Base Case Parameters

Country	Procedures, Number	SWI Events, Number	SWI Burden, US$	Mean Cost Per SWI, US$	Mean SWI Cost Per CABG, US$
France	19 280	717	8 496 817	11 845	441
Germany	50 472	2836	36 876 758	13 003	731
Italy	20 930	1378	18 940 618	13 741	905
United Kingdom	16 529	539	7 200 434	13 357	436
Netherlands	9685	814	18 365 837	22 551	1896
Spain	8294	726	8 715 780	12 008	1051
Australia	13 063	535	11 683 094	21 831	894
Israel	4037	150	2 323 092	15 508	575
Japan	21 313	1099	59 566 162	54 180	2795
Brazil	20 198	1223	9 996 520	8172	495
Canada	20 868	1239	33 091 101	26 707	1586
Taiwan	1510	103	1 467 327	14 249	971
Thailand	6581	518	4 972 120	9600	756
United States	159 063	9139	336 028 904	36 768	2113

Abbreviations: CABG, coronary artery bypass graft; SWI, sternal-wound infection.

Results are rounded to the nearest whole number. Mean SWI cost per CABG represents the SWI burden divided by the number of CABG procedures.

**Table 2:** Median Annual Burden (Range) of SWIs by Country

Country	Cost Burden, US$ in Millions	ICU Burden, Care Days	GW Burden, Care Days	Readmission Burden, Events
France	12.97 (8.32 to 27.18)	1900 (800 to 3900)	9500 (5100 to 23 500)	430 (260 to 840)
Germany	43.57 (28.06 to 81.82)	6300 (4000 to 12 500)	33 500 (20 900 to 75 500)	1450 (1050 to 2130)
Italy	20.37 (11.63 to 33.93)	3100 (1600 to 5000)	17 000 (7500 to 31 100)	600 (380 to 1030)
United Kingdom	12.34 (8.47 to 18.93)	1800 (1200 to 2800)	9800 (5800 to 15 600)	290 (200 to 400)
Netherlands	12.97 (9.24 to 21.41)	2200 (1400 to 3400)	12 300 (7800 to 19 900)	230 (150 to 320)
Spain	8.69 (4.81 to 13.24)	1200 (700 to 2200)	7000 (2900 to 12 700)	280 (150 to 380)
Australia	13.13 (6.63 to 17.35)	1900 (900 to 2800)	10 800 (4600 to 15 200)	260 (200 to 340)
Israel	3.24 (2.24 to 5.61)	600 (400 to 1000)	3400 (2200 to 5500)	70 (40 to 110)
Japan	47.25 (27.78 to 77.85)	8500 (6000 to 12 600)	50 000 (36 700 to 72 000)	320 (200 to 440)
Brazil	16.12 (9.86 to 23.33)	2900 (1800 to 4600)	16 300 (8700 to 27 500)	520 (270 to 640)
Canada	29.49 (19.42 to 50.31)	3200 (2000 to 5600)	17 300 (9000 to 34 800)	490 (340 to 710)
Taiwan	1.85 (1.29 to 3.44)	400 (600 to 300)	2300 (1300 to 3800)	40 (30 to 50)
Thailand	7.58 (3.43 to 12.58)	1400 (600 to 2400)	7900 (2800 to 15 100)	160 (120 to 220)
United States	299.40 (179.03 to 528.94)	22 600 (12 500 to 33 900)	125 100 (75 100 to 205 200)	4280 (2740 to 6100)

Abbreviations: GW, general ward; SWI, sternal-wound infection.

**Table 3:** Potential for VBP for a Hospital Performing 1000 CABG Procedures Per Year Across Analyzed Countries

Country	Scenario 1 CIPP, US$	Scenario 1 VBP, US$	Scenario 1 Hospital Savings, US$	Scenario 2 CIPP, US$	Scenario 2 VBP, US$	Scenario 2 Hospital Savings, US$
France	34 (22 to 70)	5044 (3237 to 10 574)	57 165 (36 689 to 119 839)	20 (13 to 42)	11 769 (7554 to 24 673)	70 616 (45 322 to 148 037)
Germany	43 (28 to 81)	6474 (4170 to 12 158)	73 372 (47 256 to 137 790)	26 (17 to 49)	15 106 (9729 to 28 369)	90 636 (58 375 to 170 211)
Italy	49 (28 to 81)	7298 (4168 to 12 160)	82 711 (47 240 to 137 809)	29 (17 to 49)	17 029 (9726 to 28 372)	102 173 (58 355 to 170 234)
United Kingdom	37 (26 to 57)	5599 (3841 to 8587)	63 460 (43 533 to 97 324)	22 (15 to 34)	13 065 (8963 to 20 037)	78 391 (53 776 to 120 224)
Netherlands	67 (48 to 111)	10 044 (7154 to 16 578)	113 828 (81 084 to 187 886)	40 (29 to 66)	23 435 (16 694 to 38 682)	140 611 (100 162 to 232 095)
Spain	52 (29 to 80)	7860 (4350 to 11 973)	89 077 (49 301 to 135 699)	31 (17 to 48)	18 339 (10 150 to 27 938)	110 037 (60 902 to 167 628)
Australia	50 (25 to 66)	7536 (3806 to 9959)	85 405 (43 135 to 112 873)	30 (15 to 40)	17 583 (8881 to 23 239)	105 500 (53 285 to 139 431)
Israel	40 (28 to 69)	6012 (4165 to 10 421)	68 134 (47 200 to 118 102)	24 (17 to 42)	14 028 (9718 to 24 315)	84 166 (58 306 to 145 891)
Japan	111 (65 to 183)	16 629 (9776 to 27 397)	188 460 (110 795 to 310 502)	67 (39 to 110)	38 801 (22 811 to 63 927)	232 804 (136 864 to 383 561)
Brazil	40 (24 to 58)	5987 (3662 to 8663)	67 854 (41 502 to 98 183)	24 (15 to 35)	13 970 (8545 to 20 214)	83 820 (51 267 to 121 285)
Canada	71 (47 to 121)	10 598 (6980 to 18 081)	120 108 (79 108 to 204 913)	42 (28 to 72)	24 728 (16 287 to 42 188)	148 369 (97 721 to 253 127)
Taiwan	61 (43 to 114)	9178 (6397 to 17 092)	104 017 (72 494 to 193 706)	37 (26 to 68)	21 415 (14 925 to 39 881)	128 491 (89 551 to 239 283)
Thailand	58 (26 to 96)	8644 (3905 to 14 336)	97 963 (44 256 to 162 473)	35 (16 to 57)	20 169 (9112 to 33 450)	121 013 (54 669 to 200 702)
United States	94 (56 to 166)	14 117 (8441 to 24 940)	159 992 (95 668 to 282 656)	56 (34 to 100)	32 940 (19 696 to 58 194)	197 637 (118 178 to 349 163)

Abbreviations: CABG, coronary artery bypass graft surgery; CIPP, cost of intervention per patient; SWI, sternal-wound infection; VBP, value-based purchasing.

All values are the median (range) calculated from the probabilistic sensitivity analysis results. The hospital savings assume that both new interventions implemented achieve the target of a 20% reduction in the SWI rate.

## References

[1] (2011). Report on the burden of endemic health care-associated infection worldwide.

[2] van Mourik MSM, van Duijn PJ, Moons KGM, Bonten MJM, Lee GM (2015). Accuracy of administrative data for surveillance of healthcare-associated infections: a systematic review. BMJ Open.

[3] Stone PW (2009). Economic burden of healthcare-associated infections: an American perspective. Expert Rev Pharmacoecon Outcomes Res.

[4] Arefian H, Hagel S, Heublein S (2016). Extra length of stay and costs because of health care-associated infections at a German university hospital. Am J Infect Control.

[5] World Health Organization:Regional Office for Europe (2016). Guidelines on Core Components of Infection Prevention and Control Programmes at the National and Acute Health Care Facility Level.

[6] Healthcare-associated infections.

[7] Vlaanderen FP, Tanke MA, Bloem BR (2019). Design and effects of outcome-based payment models in healthcare: a systematic review. Eur J Health Econ.

[8] Stone PW, Glied SA, McNair PD (2010). CMS changes in reimbursement for HAIs. Med Care.

[9] Hospital value-based purchasing.

[10] Hospital-Acquired Condition Reduction Program (HACRP).

[11] Hospital readmissions reduction Program (HRRP).

[12] Magid B, Murphy C, Lankiewicz J, Lawandi N, Poulton A (2018). Pricing for safety and quality in healthcare: a discussion paper. Infect Dis Health.

[13] Calderwood MS, Kleinman K, Huang SS, Murphy MV, Yokoe DS, Platt R (2017). Surgical site infections. Med Care.

[14] Bazzoli GJ, Thompson MP, Waters TM (2018). Medicare payment penalties and safety net hospital profitability: minimal impact on these vulnerable hospitals. Health Serv Res.

[15] Bai G, Anderson GF (2016). A more detailed understanding of factors associated with hospital profitability. Health Aff (Millwood).

[16] Stein SM, Day M, Karia R, Hutzler L, Bosco JA (2015). Patients’ perceptions of care are associated with quality of hospital care: a survey of 4605 hospitals. Am J Med Qual Off J Am Coll Med Qual.

[17] Isaac T, Zaslavsky AM, Cleary PD, Landon BE (2010). The relationship between patients’ perception of care and measures of hospital quality and safety. Health Serv Res.

[18] Anderson DJ, Podgorny K, Berríos-Torres SI, Bratzler DW, Dellinger EP, Greene L (2014). Strategies to prevent surgical site infections in acute care hospitals: 2014 update. Infect Control Hosp Epidemiol.

[19] Gulack BC, Kirkwood KA, Shi W (2018). Secondary surgical site infection after coronary artery bypass grafting: a multi-institutional prospective cohort study. J Thorac Cardiovasc Surg.

[20] Cotogni P, Barbero C, Rinaldi M (2015). Deep sternal wound infection after cardiac surgery: evidences and controversies. World J Crit Care Med.

[21] Greco G, Shi W, Michler RE (2015). Associated with health care-associated infections in cardiac surgery. J Am Coll Cardiol.

[22] Singh K, Anderson E, Harper JG (2011). Overview and management of sternal wound infection. Semin Plast Surg.

[23] Meszaros K, Fuehrer U, Grogg S (2016). Risk factors for sternal wound infection after open heart operations vary according to type of operation. Ann Thorac Surg.

[24] Colombier S, Kessler U, Ferrari E, von Segesser LK, Berdajs DA (2013). Influence of deep sternal wound infection on long-term survival after cardiac surgery. Med Sci Monit Int Med J Exp Clin Res.

[25] The economist intelligence unit global access to healthcare index poster 2017. The Economist Store and Economist Diaries.

[26] Saunders R, Lankiewicz J (2019). The cost effectiveness of single-patient-use electrocardiograph cable and lead systems in monitoring for coronary artery bypass graft surgery. Front Cardiovasc Med.

[27] Lankiewicz JD, Wong T, Moucharite M (2018). The relationship between a single-patient-use electrocardiograph cable and lead system and coronary artery bypass graft surgical site infection within a Medicare population. Am J Infect Control.

[28] Heart diseases 2016—Eurostat.

[29] Lee C, Cheng C, Yang YK (2014). Trends in the incidence and management of acute myocardial infarction from 1999 to 2008: get with the guidelines performance measures in Taiwan. J Am Heart Assoc Cardiovasc Cerebrovasc Dis.

[30] Hurley MP, Schoemaker L, Morton JM (2016). Geographic variation in surgical outcomes and cost between the United States and Japan. Am J Manag Care.

[31] Masuda M, Kuwano H, Okumura M (2014). Thoracic and cardiovascular surgery in Japan during 2012. Gen Thorac Cardiovasc Surg.

[32] Lonie S, Hallam J, Yii M (2015). Changes in the management of deep sternal wound infections: a 12-year review. ANZ J Surg.

[33] Zhang S, Palazuelos-Munoz S, Balsells EM, Nair H, Chit A, Kyaw MH (2016). Cost of hospital management of Clostridium difficile infection in United States—a meta-analysis and modelling study. BMC Infect Dis.

[34] Kles CL, Murrah CP, Smith K, Baugus-Wellmeier E, Hurry T, Morris CD (2015). Achieving and sustaining zero: preventing surgical site infections after isolated coronary artery bypass with saphenous vein harvest site through implementation of a staff-driven quality improvement process. Dimens Crit Care Nurs DCCN.

[35] Kieser TM, Rose MS, Aluthman U, Montgomery M, Louie T, Belenkie I (2014). Toward zero: deep sternal wound infection after 1001 consecutive coronary artery bypass procedures using arterial grafts: implications for diabetic patients. J Thorac Cardiovasc Surg.

[36] Vos RJ, Van Putte BP, Kloppenburg GTL (2018). Prevention of deep sternal wound infection in cardiac surgery: a literature review. J Hosp Infect.

[37] Schimmer C, Özkur M, Sinha B (2012). Gentamicin-collagen sponge reduces sternal wound complications after heart surgery: a controlled, prospectively randomized, double-blind study. J Thorac Cardiovasc Surg.

[38] (2010). Effect of an implantable gentamicin-collagen sponge on sternal wound infections following cardiac surgery: a randomized trial. JAMA.

[39] Bottio T, Rizzoli G, Vida V, Casarotto D, Gerosa G (2003). Double crisscross sternal wiring and chest wound infections: a prospective randomized study. J Thorac Cardiovasc Surg.

[40] Bejko J, Bottio T, Tarzia V (2015). Nitinol flexigrip sternal closure system and standard sternal steel wiring: insight from a matched comparative analysis. J Cardiovasc Med Hagerstown Md.

[41] Hamajima N, Sugimoto T, Hasebe R (2017). Medical facility statistics in Japan. Nagoya J Med Sci.

[42] Kusachi S, Kashimura N, Konishi T (2012). Length of stay and cost for surgical site infection after abdominal and cardiac surgery in Japanese hospitals: multi-center surveillance. Surg Infect.

[43] Schrijvers G, van Hoorn A, Huiskes N (2012). The care pathway: concepts and theories: an introduction. Int J Integr Care.

[44] (2015). The pros and cons of inpatient and outpatient care. Japan Today.

[45] Chawla A, Westrich K, Matter S, Kaltenboeck A, DuBois R Care pathways in US healthcare settings: current successes and limitations, and future challenges. AJMC.

