# Supplementary material for: Sternal-Wound Infections following Coronary Artery Bypass Graft: Could Implementing Value-Based Purchasing be Beneficial?
Source: J Health Econ Outcomes Res. 2020 Aug 18;7(2):130–8. doi: 10.36469/jheor.2020.13687 (PMC7458496; doi:10.36469/jheor.2020.13687)
Supplement: Supplementary file 1 [file jheor-7-2-13687-s01.pdf]

Journal of Health Economics and Outcomes Research

Supplementary Online Material

Brandt D, Blüher M, Lankiewicz J, Mallow PJ, Saunders R. Sternal-Wound Infections following Coronary Artery Bypass Graft: Could Implementing Value-Based Purchasing be Beneficial? *JHEOR.* 2020;7(2):130-138.
doi:10.36469/jheor.2020.13687

This supplementary material has been provided by the authors to give readers additional information about their work.

This is an open-access article distributed under the terms of the Creative Commons Attribution 4.0 International License (CCBY-4.0). View this license's legal deed at http://creativecommons.org/licenses/by/4.0 and legal code at http://creativecommons.org/licenses/by/4.0/legalcode for more information.

**Country Name**	**Population 100 000s**	**CABG rate Per 100 000**	**LoS Days**	**ICU cost USD per day**	**GW cost USD per day**	**SSI rate %**	**% DSWI % of SSIs**	**SSI LoS Days**	**DSWI LoS Days**
France	66.8(1)	28.9(2)	7.0(2)	1442(3)	411(4)	3.8(5)	48.0(6)	9(6)	22(6)
Germany	82.2(1)	61.4(2)	10.3(2)	1129(7)	427(4)	2.9(5)	71.6(8)	10(9)	24(10)
Italy	60.7(1)	34.5(2)	8.5(2)	1422(11)	360(4)	5.5(5)	48.0(12)	NA	30.4(13)
UK	65.4(1)	25.3(2)	11.9(2)	1582(7)	502(4)	2.0(5)	27.4(14)	11(15)	23(15)
Netherlands	17.0(1)	57.0(2)	17.8(2)	1333(7)	542(4)	10.4(16)	33.0(16)	18(16)	37(16)
Spain	46.4(1)	17.9(2)	11.1(2)	1719(7)	325(4)	5.9(17)	52.8(18)	2(19)	NA
Australia	24.3(20)	53.8(21)	10.8(22)	2617(23)	NA	3.6(24)	36.1(25)	10.8(26)	53.2(27)
Israel	8.7(20)	46.4(28)	14.1(28)	1229(29)	332(30)	3.6(31)	NA	27.9(31)	NA
Japan	127(20)	16.8(32)	34.6(33)	2130(34)	NA	4.1(35)	27.9(35)	49.1(36)	66(36)
Brazil	206.1(20)	9.8(37)	11.2(38)	1143(39)	142(40)	5.1(41)	36.4(41)	NA	18(42)
Canada	36.2(20)	57.6(43)	13.4(44)	4020(44)	1270 (44)	4.6(45–47)	38.9(45,46)	9.6(46,47)	NA
Taiwan	23.6(20)	6.4(48)	20.8(49)	1041(50)	226(49)	4.3(49)	50(49)	32.2(51)	NA
Thailand	69.3(20)	9.5(52)	12.6(52)	690(53)	NA	7.9(54)	10(54)	NA	NA
US	323.3(20)	49.2(55)	8.9(55)	4576(56)	2446(57)	4.4(58–63)	46(58–60,62–65)	13.5(58,61)	25.5(58,64)

NA: Not Available

1. Eurostat. Population statistics. 2019;
2. Eurostat. Cardiovascular diseases statistics. 2016;
3. Lefrant J-Y, Garrigues B, Pribil C, Bardoulat I, Courtial F, Maurel F, et al. The daily cost of ICU patients: A micro-costing study in 23 French Intensive Care Units. Anaesthesia, critical care & pain medicine. 2015 Jun;34(3):151–7.
4. World Health Organization (WHO). Country-specific unit costs. 2008;
5. European Centre for Disease Prevention and Control. Healthcare-associated infections: surgical site infections. ECDC Annual epidemiological report for 2016 Stockholm: ECDC. 2018;(May).
6. Cossin S, Malavaud S, Jarno P, Giard M, L'Hériteau F, Simon L, et al. Surgical site infection after valvular or coronary artery bypass surgery: 2008-2011 French SSI national ISO-RAISIN surveillance. Journal of Hospital Infection. 2015;91(3):225–30.
7. Bittner MI, Donnelly M, van Zanten ARH, Andersen JS, Guidet B, Cabello JJT, et al. How is intensive care reimbursed? A review of eight European countries. Annals of Intensive Care. 2013;3(1):1–9.
8. KISS Hospital Infection Surveillance System (. 2010;(January 2005).
9. Gorlitzer M, Wagner F, Pfeiffer S, Folkmann S, Meinhart J, Fischlein T, et al. Prevention of sternal wound complications after sternotomy: Results of a large prospective randomized multicentre trial. Interactive Cardiovascular and Thoracic Surgery. 2013;17(3):515–22.
10. Graf K, Ott E, Vonberg RP, Kuehn C, Haverich A, Chaberny IF. Economic aspects of deep sternal wound infections. European Journal of Cardio-thoracic Surgery. 2010;37(4):893–6.
11. Tan SS, Bakker J, Hoogendoorn ME, Kapila A, Martin J, Pezzi A, et al. Direct cost analysis of intensive care unit stay in four European countries: applying a standardized costing methodology. Value in health : the journal of the International Society for Pharmacoeconomics and Outcomes Research. 2012 Jan;15(1):81–6.
12. De Santo LS, Bancone C, Santarpino G, Romano G, De Feo M, Scardone M, et al. Microbiologically documented nosocomial infections after cardiac surgery: an 18-month prospective tertiary care centre report. European Journal of Cardio-thoracic Surgery. 2008;33(4):666–72.
13. D'Agostino D, Man A, Santacroce L. Current trends in cardiac surgery: Clinical experience in the treatment of mediastinitis with sternal wound infection through negative pressure therapy. Acta Medica Mediterranea. 2016;32(5):195–201.
14. Ariyaratnam P, Bland M, Loubani M. Risk factors and mortality associated with deep sternal wound infections following coronary bypass surgery with or without concomitant procedures in a UK population: a basis for a new risk model? Interactive CardioVascular and Thoracic Surgery. 2010;11(5):543–6.
15. Jenks PJ, Laurent M, McQuarry S, Watkins R. Clinical and economic burden of surgical site infection (SSI) and predicted financial consequences of elimination of SSI from an English hospital. Journal of Hospital Infection. 2014;86(1):24–33.
16. van der Slegt J, van der Laan L, Veen EJ, Hendriks Y, Romme J, Kluytmans J. Implementation of a Bundle of Care to Reduce Surgical Site Infections in Patients Undergoing Vascular Surgery. PLoS ONE. 2013;8(8):1–7.
17. ECDC. Surveillance of surgical site infections in Europe. 2013.
18. Tamayo E, Gualis J, Flórez S, Castrodeza J, Eiros Bouza JM, Álvarez FJ. Comparative study of single-dose and 24-hour multiple-dose antibiotic prophylaxis for cardiac surgery. Journal of Thoracic and Cardiovascular Surgery. 2008;136(6):1522–7.
19. Figuerola-Tejerina A, Rodríguez-Caravaca G, Bustamante-Munguira J, María San Román-Montero J, Durán-Poveda M. Epidemiological Surveillance of Surgical Site Infection and its Risk Factors in Cardiac Surgery: A Prospective Cohort Study. Revista Española de Cardiología (English Edition). 2016;69(9):842–8.
20. Population by Country (2019) - Worldometers [Internet]. [cited 2019 Dec 8]. Available from: https://www.worldometers.info/world-population/population-by-country/
21. OECD. Cardiac procedures. 2015 Nov 4;110–1.
22. Oxlad M, Stubberfield J, Stuklis R, Edwards J, Wade TD. Psychological Risk Factors for Increased Post-Operative Length of Hospital Stay Following Coronary Artery Bypass Graft Surgery. J Behav Med. 2006 Feb 22;29(2):179.
23. Intensive care unit costings : Anaesthesia and Intensive Care Journal [Internet]. [cited 2019 Dec 8]. Available from: https://aaic.net.au/Document/?D=2004260
24. Si D, Rajmokan M, Lakhan P, Marquess J, Coulter C, Paterson D. Surgical site infections following coronary artery bypass graft procedures: 10 years of surveillance data. BMC Infect Dis. 2014 Jun 10;14:318.
25. Saxena A, Dinh D, Smith JA, Shardey G, Reid CM, Newcomb AE. Sex differences in outcomes following isolated coronary artery bypass graft surgery in Australian patients: analysis of the Australasian Society of Cardiac and Thoracic Surgeons cardiac surgery database. Eur J Cardiothorac Surg. 2012 Apr;41(4):755–62.
26. Jenney AWJ, Harrington GA, Russo PL, Spelman DW. Cost of surgical site infections following coronary artery bypass surgery. ANZ Journal of Surgery. 2001;71(11):662–4.
27. Lonie S, Hallam J, Yii M, Davis P, Newcomb A, Nixon I, et al. Changes in the management of deep sternal wound infections: a 12-year review. ANZ J Surg. 2015 Nov;85(11):878–81.
28. Blumenfeld Orit, Na'amnih Wasef, Shapira-Daniels Ayelet, Lotan Chaim, Shohat Tamy, Shapira Oz M. Trends in Coronary Revascularization and Ischemic Heart Disease–Related Mortality in Israel. Journal of the American Heart Association. 6(2):e004734.

29. Ginsberg GM, Kark JD, Einav S. Cost–utility analysis of treating out of hospital cardiac arrests in Jerusalem. Resuscitation. 2015 Jan 1;86:54–61.

30. WHO | Israel [Internet]. WHO. [cited 2019 Dec 8]. Available from: https://www.who.int/choice/country/isr/cost/en/

31. Paul M, Raz A, Leibovici L, Madar H, Holinger R, Rubinovitch B. Sternal wound infection after coronary artery bypass graft surgery: Validation of existing risk scores. The Journal of Thoracic and Cardiovascular Surgery. 2007 Feb 1;133(2):397–403.

32. Masuda M, Kuwano H, Okumura M, Amano J, Arai H, Endo S, et al. Thoracic and cardiovascular surgery in Japan during 2012. Gen Thorac Cardiovasc Surg. 2014;62:734–64.

33. Geographic Variation in Surgical Outcomes and Cost Between the United States and Japan [Internet]. AJMC. [cited 2019 Dec 8]. Available from: https://www.ajmc.com/journals/issue/2016/2016-vol22-n9/geographic-variation-in-surgical-outcomes-and-cost-between-the-united-states-and-japan

34. Cao P, Toyabe S, Abe T, Akazawa K. Profit and loss analysis for an intensive care unit (ICU) in Japan: a tool for strategic management. BMC Health Services Research. 2006 Jan 11;6(1):1.

35. Morikane K, Honda H, Yamagishi T, Suzuki S. Differences in risk factors associated with surgical site infections following two types of cardiac surgery in Japanese patients. J Hosp Infect. 2015 May;90(1):15–21.

36. Kobayashi J, Kusachi S, Sawa Y, Motomura N, Imoto Y, Makuuchi H, et al. Socioeconomic effects of surgical site infection after cardiac surgery in Japan. Surg Today. 2015 Apr;45(4):422–8.

37. Gomes WJ, Moreira RS, Zilli AC, Jr LCB, Figueira FAM dos S, D'Azevedo SSP, et al. The Brazilian Registry of Adult Patient Undergoing Cardiovascular Surgery, the BYPASS Project: Results of the First 1,722 Patients. Brazilian Journal of Cardiovascular Surgery. 2017 Apr;32(2):71.

38. Silva GS da, Colósimo FC, Sousa AG de, Piotto RF, Castilho V, Silva GS da, et al. Coronary Artery Bypass Graft Surgery Cost Coverage by the Brazilian Unified Health System (SUS). Brazilian Journal of Cardiovascular Surgery. 2017 Aug;32(4):253–9.

39. Quintano Neira RA, Hamacher S, Japiassú AM. Epidemiology of sepsis in Brazil: Incidence, lethality, costs, and other indicators for Brazilian Unified Health System hospitalizations from 2006 to 2015. PLoS One [Internet]. 2018 Apr 13 [cited 2019 Dec 9];13(4). Available from: https://www.ncbi.nlm.nih.gov/pmc/articles/PMC5898754/

40. WHO | Brazil [Internet]. WHO. [cited 2019 Dec 9]. Available from: https://www.who.int/choice/country/bra/cost/en/

41. Ledur P, Almeida L, Pellanda LC, Schaan BD. Predictors of infection in post-coronary artery bypass graft surgery. Brazilian Journal of Cardiovascular Surgery. 2011 Jun;26(2):190–6.

42. Horácio G de S, Coltro PS, Albacete Neto A, Almeida JB, Silva VZ da, Almeida I de R, et al. Application of Unilateral Pectoralis Major Muscle Flap in the Treatment of Sternal Wound Dehiscence. Brazilian Journal of Cardiovascular Surgery. 2017 Oct;32(5):378–82.

43. Health at a Glance 2015: OECD Indicators [Internet]. [cited 2019 Dec 9]. Available from: https://www.oecd-ilibrary.org/social-issues-migration-health/health-at-a-glance-2015_health_glance-2015-en

44. Care in Canadian ICUs. :36.

45. Mocanu V, Buth KJ, Johnston LB, Davis I, Hirsch GM, Légaré J-F. The Importance of Continued Quality Improvement Efforts in Monitoring Hospital-Acquired Infection Rates: A Cardiac Surgery Experience. Ann Thorac Surg. 2015 Jun;99(6):2061–9.

46. Iribarne A, Chang H, Alexander JH, Gillinov AM, Moquete E, Puskas JD, et al. Readmissions After Cardiac Surgery: Experience of the NIH / CIHR Cardiothoracic Surgical Trials Network. Ann Thorac Surg. 2014 Oct;98(4):1274–80.

47. Shehata N, Forster A, Li L, Rothwell DM, Mazer CD, Naglie G, et al. Does anemia impact hospital readmissions after coronary artery bypass surgery? Transfusion. 2013 Aug;53(8):1688–97; quiz 1687.

48. Lee C, Cheng C, Yang YK, Chao T, Chen J, Liu P, et al. Trends in the Incidence and Management of Acute Myocardial Infarction From 1999 to 2008: Get With the Guidelines Performance Measures in Taiwan. J Am Heart Assoc [Internet]. 2014 Aug 11 [cited 2019 Dec 8];3(4). Available from: https://www.ncbi.nlm.nih.gov/pmc/articles/PMC4310397/

49. Yu T-H, Tung Y-C, Chung K-P. Does Categorization Method Matter in Exploring Volume-Outcome Relation? A Multiple Categorization Methods Comparison in Coronary Artery Bypass Graft Surgery Surgical Site Infection. Surg Infect (Larchmt). 2015 Aug;16(4):466–72.

50. Cheng K-C, Lu C-L, Chung Y-C, Huang M-C, Shen H-N, Chen H-M, et al. ICU service in Taiwan. Journal of Intensive Care. 2014 Feb 5;2(1):8.

51. Chien C-Y, Lin C-H, Hsu R-B. Care bundle to prevent methicillin-resistant Staphylococcus aureus sternal wound infection after off-pump coronary artery bypass. American Journal of Infection Control. 2014 May 1;42(5):562–4.

52. Sakornpant P, Kojaranjit V. Database overview [Internet]. The Society of Thoracic Surgeons of Thailand; 2011 [cited 2019 Dec 8]. Available from: https://www.ncbi.nlm.nih.gov/books/NBK154624/

53. Cost of Critically Ill Surgical Patients in Thailand: A Prospective Analysis of a Multicenter THAI-SICU Study [Internet]. [cited 2019 Dec 8]. Available from: https://www.researchgate.net/publication/309209410_Cost_of_Critically_Ill_Surgical_Patients_in_Thailand_A_Prospective_Analysis_of_a_Multicenter_THAI-SICU_Study

54. Sutthiruk N, Botti M, Considine J, Driscoll A, Hutchinson A, Malathum K, et al. Abstracts from the 8th International Congress of the Asia Pacific Society of Infection Control (APSIC): Bangkok, Thailand. 12-15 February 2017. Antimicrob Resist Infect Control [Internet]. 2017 [cited 2019 Dec 8];6(Suppl 2). Available from: https://europepmc.org/articles/PMC5333188/

55. HCUPnet [Internet]. HCUPnet. [cited 2019 Dec 9]. Available from: https://hcupnet.uat.s-3.net

56. Dasta JF, McLaughlin TP, Mody SH, Piech CT. Daily cost of an intensive care unit day: the contribution of mechanical ventilation. Crit Care Med. 2005 Jun;33(6):1266–71.

57. Hospital Adjusted Expenses per Inpatient Day [Internet]. The Henry J. Kaiser Family Foundation. 2019 [cited 2019 Dec 9]. Available from: https://www.kff.org/health-costs/state-indicator/expenses-per-inpatient-day/

58. Lemaignen A, Birgand G, Ghodhbane W, Alkhoder S, Lolom I, Belorgey S, et al. Sternal wound infection after cardiac surgery: incidence and risk factors according to clinical presentation. Clin Microbiol Infect. 2015 Jul;21(7):674.e11-18.

59. Huang SS, Placzek H, Livingston J, Ma A, Onufrak F, Lankiewicz J, et al. Use of Medicare claims to rank hospitals by surgical site infection risk following coronary artery bypass graft surgery. Infect Control Hosp Epidemiol. 2011 Aug;32(8):775–83.

60. Berríos-Torres SI, Mu Y, Edwards JR, Horan TC, Fridkin SK. Improved risk adjustment in public reporting: coronary artery bypass graft surgical site infections. Infect Control Hosp Epidemiol. 2012 May;33(5):463–9.

61. Coronary artery bypass graft readmission rates and risk factors - A retrospective cohort study. - PubMed - NCBI [Internet]. [cited 2019 Dec 9]. Available from: https://www.ncbi.nlm.nih.gov/pubmed/29678620

62. Itagaki S, Cavallaro P, Adams DH, Chikwe J. Bilateral internal mammary artery grafts, mortality and morbidity: an analysis of 1 526 360 coronary bypass operations. Heart. 2013 Jun;99(12):849–53.

63. Williams JB, Peterson ED, Brennan JM, Sedrakyan A, Tavris D, Alexander JH, et al. Association between endoscopic vs open vein-graft harvesting and mortality, wound complications, and cardiovascular events in patients undergoing CABG surgery. JAMA. 2012 Aug 1;308(5):475–84.

64. Sears ED, Momoh AO, Chung KC, Lu Y-T, Zhong L, Waljee JF. A National Study of the Impact of Delayed Flap Timing for Treatment of Patients with Deep Sternal Wound Infection. Plast Reconstr Surg. 2017 Aug;140(2):390–400.

65. Likosky DS, Wallace AS, Prager RL, Jacobs JP, Zhang M, Harrington SD, et al. Sources of Variation in Hospital-level Infection Rates after Coronary Artery Bypass Grafting: An analysis of The Society of Thoracic Surgeons Adult Heart Surgery Database. Ann Thorac Surg. 2015 Nov;100(5):1570–6.
